# Serotonergic innervation and serotonin receptor expression of NPY-producing neurons in the rat lateral and basolateral amygdaloid nuclei

**DOI:** 10.1007/s00429-012-0406-5

**Published:** 2012-04-17

**Authors:** M. Bonn, A. Schmitt, K.-P. Lesch, E. J. Van Bockstaele, E. Asan

**Affiliations:** 1 Institute of Anatomy and Cell Biology, University of Wuerzburg, Koellikerstr. 6, 97070 Würzburg, Germany; 2 Molecular Psychiatry, Department of Psychiatry, Psychosomatics and Psychotherapy, University of Würzburg, Fuechsleinstr. 15, 97080 Würzburg, Germany; 3 Department of Neuroscience, Farber Institute for Neurosciences, Thomas Jefferson University, Philadelphia, PA 19107 USA; 4 Department of Psychiatry, Psychosomatics and Psychotherapy, University of Würzburg, Fuechsleinstr. 15, 97080 Würzburg, Germany

**Keywords:** Anxiety, Amygdala, Serotonergic system, NPY

## Abstract

Pharmacobehavioral studies in experimental animals, and imaging studies in humans, indicate that serotonergic transmission in the amygdala plays a key role in emotional processing, especially for anxiety-related stimuli. The lateral and basolateral amygdaloid nuclei receive a dense serotonergic innervation in all species studied to date. We investigated interrelations between serotonergic afferents and neuropeptide Y (NPY)-producing neurons, which are a subpopulation of inhibitory interneurons in the rat lateral and basolateral nuclei with particularly strong anxiolytic properties. Dual light microscopic immunolabeling showed numerous appositions of serotonergic afferents on NPY-immunoreactive somata. Using electron microscopy, direct membrane appositions and synaptic contacts between serotonin-containing axon terminals and NPY-immunoreactive cellular profiles were unequivocally established. Double in situ hybridization documented that more than 50 %, and about 30–40 % of NPY mRNA-producing neurons, co-expressed inhibitory 5-HT1A and excitatory 5-HT2C mRNA receptor subtype mRNA, respectively, in both nuclei with no gender differences. Triple in situ hybridization showed that individual NPY mRNA-producing interneurons co-express both 5-HT1A and 5-HT2C mRNAs. Co-expression of NPY and 5-HT3 mRNA was not observed. The results demonstrate that serotonergic afferents provide substantial innervation of NPY-producing neurons in the rat lateral and basolateral amygdaloid nuclei. Studies of serotonin receptor subtype co-expression indicate a differential impact of the serotonergic innervation on this small, but important, population of anxiolytic interneurons, and provide the basis for future studies of the circuitry underlying serotonergic modulation of emotional stimulus processing in the amygdala.

## Introduction

The amygdala is a heterogeneous telencephalic nuclear complex which plays a key role in the processing of emotional, particularly innate anxiety- and fear-related stimuli, including the mediation of adequate autonomous, endocrine and behavioral reactions, and the formation of emotional memories both in rodents and humans (Damsa et al. 2009; LeDoux 2000; Roozendaal et al. 2009). Each amygdaloid nucleus subserves certain functions in emotion processing. Thus, the lateral nucleus (La) is the amygdala’s “gatekeeper”, receiving input from different sensory systems. From there, information is, in part, further transferred to the basolateral nucleus (BL), which additionally receives hippocampal input. From La and BL, projections to the central nucleus (Ce) arise, which is the most important output station of the amygdala to endocrine and autonomous centers in the hypothalamus and brainstem (Ehrlich et al. 2009; LeDoux 2000, 2007; Pitkanen et al. 1997).

Morphological and functional studies of the last decade have yielded strong evidence that it is activation of the amygdala, in particular, which underlies enhanced anxiety (LeDoux 2000; Roozendaal et al. 2009). In addition, functional imaging studies in humans consistently show pathological activity patterns of the amygdala during the course of affective disorders (Etkin and Schatzberg 2011). Findings in experimental animals indicate that increased neuronal activity, particularly of the basolateral amygdala, is correlated with increased anxiety-like behavior (Wang et al. 2011). Amygdala hyperexcitability accompanied by increased spine density of pyramidal neurons in these nuclei after stress has been found in wild type and genetically modified mouse models for anxiety-related neuropsychiatric disorders (Nietzer et al. 2011; Rosenkranz et al. 2010). Pyramidal cells constitute about 85 % of the neuronal population of the cortex-like La and BL. Electrophysiological studies indicate that their activity is tightly controlled by local inhibitory interneurons (Rainnie et al. 1991; Washburn and Moises 1992), which are mostly GABAergic and can be classified into different subpopulations based on the additional expression of calcium-binding proteins and/or neuropeptides (Muller et al. 2003, 2007a). Neuropeptide Y (NPY)-immunoreactive (ir) interneurons, which are a subgroup of Calbindin (CB)- and somatostatin (SOM)-ir interneurons (McDonald 1989, Truitt et al. 2009) are thought to be involved in anxiolytic actions in the La and BL of rodents and humans. Thus, electrophysiology studies documented significant inhibitory actions of NPY on La pyramidal cell activity (Sosulina et al. 2008). Moreover, in rats, the number of NPY-ir neurons in La and BL is correlated inversely with anxiety-like behavior (Yilmazer-Hanke et al. 2002, 2004), and lesion of NPY-ir neurons results in increased anxiety-like behavior (Truitt et al. 2009). In humans, a 30 % decrease in NPY mRNA expression due to a genetic polymorphism within the human *NPY* promoter region is associated with increased amygdala activity upon exposure to threat-related facial expressions (Zhou et al. 2008).

In all species studied to date, the La and BL possess an exceptionally dense serotonergic innervation arising mainly from the dorsal raphe nucleus (DR) (Fallon and Ciofi 1992; Smith and Porrino 2008). Serotonergic neurotransmission is implicated in various functions in the central nervous system, ranging from the regulation of food intake, body temperature and biorhythms to influencing attention, motivation and other cognition (Kriegebaum et al. 2010). One of its most relevant functions, from a psychiatric and social point of view, is its impact on emotional states like anxiety and associated psychiatric diseases. Thus, altered activation of the amygdala during the processing of emotional stimuli is characteristically observed in human carriers of the low-expressing variant of the serotonin transporter gene (*5*-*HTT)*, a common polymorphism which is associated with anxiety-related traits and an increased risk to develop post-traumatic disorder or depression following stress experience (Caspi et al. 2003; Grabe et al. 2005; Hariri et al. 2002; Lesch et al. 1996). There is ample electrophysiological and pharmacobehavioral evidence that alterations in serotonergic neurotransmission or differential serotonin receptor (5-HTR) activation in the amygdala impact anxiety-like behavior in rodents (Holmes 2008; Lowry et al. 2005). The serotonin receptors 1A (5-HT1A), 2C (5-HT2C) and 3 (5-HT3) are expressed in the La and BL (Clemett et al. 2000; Miquel et al. 2002; Pazos and Palacios 1985) and seem to be crucially involved in the regulation of anxiety-related neuronal circuitries. These findings render interactions of the serotonergic system with amygdaloid circuits of significant interest for emotion research, and numerous pharmacobehavioral studies have been carried out to elucidate effects of serotonergic agonist or antagonist application into the amygdala, albeit with occasionally controversial results (Barnes and Sharp 1999; Lowry et al. 2005; Menard and Treit 1999).

Interpretation of these results requires identification of serotonergic target neurons and of their receptor complement. In previous electron microscopic studies, membrane appositions and synaptic contacts of serotonergic afferents were verified on dendritic spines and distal dendrites of calcium/calmodulin-dependent protein kinase II (CaMKII)-ir pyramidal cells and on parvalbumin (PV)-ir and vasoactive intestinal polypeptide (VIP)-ir interneurons of the basolateral amygdaloid nucleus (Muller et al. 2007b). Moreover, 5-HTR expression has been documented in different neuron types (Aznar et al. 2003; Liu et al. 2007; Mascagni and McDonald 2007; Yuen et al. 2008). Light microscopic analyses additionally indicated contacts between serotonergic afferents and, for instance, CB- and SOM-ir interneurons (Muller et al. 2007b). Although of obvious interest, analyses of a possible serotonergic innervation of NPY-producing interneurons have not been carried out yet. Therefore, in the present study, we performed dual immunolabeling on a light (LM) and electron microscopic (EM) level and correlative in situ hybridization (ISH) for 5-HTR mRNA expression to determine interrelations between the serotonergic system and the unique and special interneuron subpopulation of anxiolytic NPY-producing neurons in the La and BL. Since central serotonin levels differ between male and female rats (Rosecrans 1970) and behavioral studies in rodents suggest that changes in 5-HTR equipment differentially affect behavior of males and females (Bhatnagar et al. 2004), 5-HTR expression studies were performed separately in male and female rats.

## Experimental procedures

### Tissue preparation for LM, EM and ISH

Brains of 3-month-old male and female Wistar rats (Charles River, Sulzfeld, Germany or Philadelphia, PA, USA) were used for all experiments. Animal experiments were carried out according to the German Law for the Protection of Animals or were approved by the Institutional Animal Care and Use Committee (IACUC) of Thomas Jefferson University and were conducted in accordance with the NIH *Guide for the care and use of laboratory animals*. Only the minimal number of animals necessary to produce reliable scientific data was used, and all efforts were made to minimize animal suffering. For tissue preparation for LM immunohistochemical studies, anesthetized rats were briefly perfused via the left ventricle with 0.7 % heparin in 0.9 % NaCl followed by perfusion with either (1) freshly prepared 4 % formaldehyde (FA) in 0.01 M phosphate buffered saline (PBS, pH 7.4; female rats, *n* = 4) or (2) FA fixatives at variable pH (female rats, *n* = 8) as described previously (Asan 1998; Eliava et al. 2003). The brains were then dissected and post-fixed over night at 4 °C in the corresponding fixative [without glutaraldehyde in (2)]. Subsequently, tissue was successively infiltrated with 10 and 20 % sucrose in PBS, frozen in liquid nitrogen-cooled 2-methylbutane and stored at −80 °C. For sectioning, tissue was slowly thawed to 4 °C and serial 45-μm-thick coronal vibratome sections were cut in a PBS bath at 4 °C (Eliava et al. 2003).

For EM, female (*n* = 2) and male (*n* = 2) anesthetized rats were pre-rinsed with a heparin-sodium solution, perfused using freshly prepared 4 % FA in 0.1 M phosphate buffer (PB, pH 7.4) and post-fixed in the same fixative over night at 4 °C. Then, coronal sections (40 μm) were cut on a vibratome in 0.1 M PB at room temperature and subsequently used for EM. Vibratome sections for EM and LM were processed for immunohistochemistry in wells of tissue culture plates.

For ISH, female (*n* = 7) and male (*n* = 9) anesthetized rats were decapitated, brains were immediately dissected, frozen in liquid nitrogen-cooled 2-methylbutane and stored at −80 °C. Serial coronal sections (10 μm) were cut on a cryostat set at −25 °C and thaw-mounted on slides. Subsequently, sections were fixed in freshly prepared 4 % FA in 0.01 M PBS, transferred to 100 % ethanol and stored at 4 °C as described previously (Bonn et al. 2012). If not mentioned otherwise, the following steps were carried out at room temperature.

### Single and double immunohistochemistry for LM

Vibratome sections were pre-incubated in 5 % normal goat serum (NGS) diluted in 0.5 % Triton X-100 in 0.01 M PBS for 2 h and subsequently incubated in antibody solution consisting of PBS with 0.5 % Triton X-100 and 1 % NGS as described by Eliava et al. (2003). Serotonergic fibers were detected using polyclonal rabbit (rb) anti-5-HTT (1:750, catalog #PC177L, Merck, Darmstadt, Germany) or polyclonal rb anti-serotonin (5-HT, 1:45,000, catalog #20080, Sorin Biomedica, Duesseldorf, Germany). Primary antibody incubation was done for 48 h at 4 °C with light agitation. Then, sections were washed 6× 10 min in PBS and incubated over night at 4 °C with biotinylated goat anti-rb IgG (1:500, catalog #BA-1000 Vector, Wiesbaden, Germany), followed by incubation in avidin–biotin complex (ABC) for 2 h according to Eliava and co-workers (2003). Chromogenic visualization was carried out applying the glucose oxidase-diaminobenzidine method described by Zaborszky and Heimer (1989). This immunoreaction was amplified by nickel intensification (Liposits et al. 1986). For double labeling, the sections were further reacted with the second primary antibody (polyclonal rb anti-NPY, 1:8000, catalog #N9528, Sigma, Munich, Germany), biotinylated goat anti-rb IgG and ABC as described above and the NPY signal was visualized with non-intensified DAB (Eliava et al. 2003). After rinsing in three changes of PBS (10 min each), sections were mounted on glass slides, dried overnight, dehydrated in ethanol (70, 90, 96, 2× 100 %, 3 min each), cleared in xylene (2× 3 min) and coverslipped in DEPEX (Serva, Heidelberg, Germany). Sections were analyzed with a Zeiss axioscope (Zeiss, Oberkochen, Germany) and photographs were made using the Neurolucida system (Microbrightfield, Magdeburg, Germany). Pictures were adjusted for brightness and contrast in Adobe Photoshop CS.

### Double immunohistochemistry for EM

Vibratome sections were rinsed in 0.1 M Tris-saline buffer (TS, pH 7.6, 2× 10 min), incubated in TS containing 0.5 % BSA for 30 min and briefly rinsed in TS again. Then, sections were incubated for 46–48 h in an antibody cocktail of polyclonal rb anti-NPY (1:8000, see above) and polyclonal rat anti-5-HT (1:500, catalog #MAS055, Harlan-Sera-Lab, Belton, United Kingdom) diluted in TS containing 0.1 % BSA. The following steps were all carried out according to Waselus and co-workers (2005): (1) incubation with biotinylated goat anti-rb secondary antibody (1:500, catalog #BA-1000, Vector Laboratories, Burlingame, CA) and ABC (Vector Laboratories), (2) visualization of NPY with DAB, detection of 5-HT immunoreactivity by immunogold-silver localization using goat anti-rat IgG conjugated to 1-nm gold particles (1:50, catalog #25181, Electron Microscopy Sciences, Fort Washington, PA, USA), (3) incubation in 2 % glutaraldehyde in 0.01 M PBS for 10 min, (4) silver intensification of gold particles for 10 min using a silver enhancement kit (Polysciences Inc., Warrington, PA), (5) incubation in 2 % osmium tetroxide in 0.1 M PB for 1 h, (6) dehydration and (7) flat embedding. Then, a small area containing La and BL was cut from the flat embedded section and re-embedded onto Epon blocks. Ultrathin sections (74 nm) containing La or BL, were cut, placed onto grids, contrasted with uranyl acetate and lead citrate (Reynolds 1963) and examined with an electron microscope (LEO AB 912, Zeiss NTS, Oberkochen, Germany and Morgagni, Fei Company, Hillsboro, OR). Pictures were adjusted for brightness and contrast in Adobe Photoshop CS.

### In situ hybridization

Generation of cRNA probes specific for rat NPY (GenBank accession: NM_012614), rat 5-HT1A (GenBank accession: NM_012585.1) and rat 5-HT2C mRNA (GenBank accession: NM_012765.3) was performed as described by Bonn et al. (2012). A cDNA fragment of mouse 5-HT3 mRNA (GenBank accession: NM_013561.2; 92 % homology to the corresponding rat sequence) was cloned into pGEM-T vector (Promega, Mannheim, Germany) and further processed for generation of antisense and sense cRNA probes as previously described (Bonn et al. 2012).

Procedures for hapten labeling of cRNA probes were done as follows: for single labelings, cRNAs were reacted using biotin-, fluorescein- or digoxigenin (DIG)-RNA labeling mixes (all Roche, Mannheim, Germany). For double labelings, fluorescein- and DIG-labeled cRNA probes were used for NPY and 5-HT2C mRNA detection as required; for 5-HT1A mRNA detection only DIG-labeled cRNA was used. For triple labelings, NPY cRNA was reacted using biotin-RNA labeling mix, 5-HT2C cRNA with fluorescein-RNA labeling mix and 5-HT1A cRNA with DIG-RNA labeling mix. Rehydration in a graded series of ethanol, acetylation and pre-hybridization were done according to Bonn et al. (2012). Sections were covered with hybridization solution containing one, two or three differently labeled cRNA probes of interest. Hybridization to nucleic acid targets was carried out for 16–20 h at 57–58 °C followed by post-hybridization washes as described by Bonn et al. (2012). Subsequently, tissue was treated with blocking buffer according to the manufacturer’s instruction (Perkin Elmer, Rodgau, Germany). ISH signals were detected using either of four methodic variations: (1) for chromogenic detection (CISH), blocked sections were incubated in alkaline phosphatase (AP)-conjugated anti-fluorescein (1:600, Roche) or anti-DIG Fab fragments (1:600, Roche) for 2.5 h, rinsed and subjected to chromogenic detection with nitro blue tetrazolium (NBT)/5-bromo-4-chloro-3′-indolyphosphate (BCIP; Roche) for 16–20 h. Development of the enzyme reaction was carried out in the dark and chromogenic signal intensity was controlled by brief microscopic observation at regular intervals. (2) For highly sensitive tyramide signal amplified fluorescence ISH (HS-TSA-FISH), sections were incubated in horseradish peroxidase (POD)-conjugated anti-DIG or anti-fluorescein Fab fragments (1:1000, Roche) for 2.5 h, reacted with TSA™ Biotin (Perkin Elmer) and further incubated in the dark with AlexaFluor^®^ 488-conjugated streptavidin (Invitrogen, Darmstadt, Germany). (3) For moderately sensitive TSA fluorescence ISH (MS-TSA-FISH), tissue incubated with POD-conjugated Fab fragments as described above was reacted with TSA™ Cy3 (Perkin Elmer). (4) For low sensitive TSA fluorescence ISH (LS-TSA-FISH), sections reacted with a biotin-labeled cRNA probe were incubated with POD-conjugated streptavidin (Perkin Elmer) rinsed and reacted with TSA™ Cy3 (for further details see Bonn et al. 2012).

Chromogenic detection-reacted sections were mounted in Aquatex^®^ (Merck), in FISH-reacted sections nuclei were stained using 300 nM DAPI (Roche) and sections were mounted with Fluoro-Gel (Science Services, Munich, Germany). With single CISH, clear and specific signals were produced for each cRNA probe. Equivalent reaction intensity was found for NPY and 5-HT2C mRNA single detection using HS-TSA-FISH and for NPY also using MS-TSA-FISH. NPY mRNA detection using LS-TSA-FISH labeled cells less intensely and was therefore not used for quantitative analyses (not shown; cf Bonn et al. 2012). For NPY/5-HT2C double FISH, first HS-TSA-FISH was applied to detect 5-HT2C mRNA. Reacted sections were then subjected to a POD block in 0.02 N HCl, rinsed and then MS-TSA-FISH was applied to detect NPY mRNA. For detection of NPY with 5-HT1A and with 5-HT3 mRNA, respectively, a combination of FISH and CISH previously described by Bonn et al. (2012) was applied: the fluorescein-labeled NPY cRNA was first detected using HS-TSA-FISH followed by CISH detection of DIG-labeled receptor cRNA probes, DAPI-staining and mounting. For triple labelings (NPY, 5-HT1A and 5-HT2C mRNA), we applied double FISH using HS-TSA-FISH for 5-HT2C and LS-TSA-FISH for NPY mRNA detection, respectively, followed by CISH for 5-HT1A mRNA detection, DAPI-staining and mounting. Sections were observed with a Zeiss axioscope (see above) equipped with appropriate fluorescence and bright field filter systems and analyzed using a digital Spot camera (Visitron Systems, Puchheim, Germany) and the Spot Advanced software (Diagnostic Instruments, Inc., Sterling Heights, MI, USA). Pictures were adjusted for brightness and contrast, merged and assembled in Spot Advanced and Adobe Photoshop CS.

### Analysis and controls

On the LM level, La and BL were identified according to the rat brain atlas by Paxinos and Watson (2007) at low magnification, all NPY-ir neurons were localized and counted and the number of apparent serotonergic contacts was determined. Each 5-HTT-ir fiber apparently contacting an NPY-ir soma (no visible gaps between fiber and NPY-ir soma at different focus levels at 100× magnification with immersion oil) was considered as one apposition. For each animal, we analyzed four to eight vibratome sections containing La and BL at anterior, medial and posterior coronal section level. For controls, we omitted the primary antibodies from the first, second or from both reaction sequences.

To ensure that both immunolabels were clearly detectable in ultrathin sections used for EM investigations, only tissue sections collected near the tissue-Epon interface, where penetration of the antibodies was optimal, were included in the analyses. NPY-ir structures were identified due to intense DAB labeling. Synaptic axon terminals were only counted as 5-HT-ir if at least two immunogold-silver particles were present and if the same structure displayed labeling in several serial sections. To ensure specificity of the immunogold-silver signal, the surrounding neuropil was analyzed and it was established that no unspecific labeling, e.g. in myelin sheath, was present. When primary antibodies were omitted in controls, labeling was lacking in both DAB and immunogold silver-stained sections.

In ISH control labelings, substitution of antisense cRNA probes by an equivalent amount of hapten-labeled sense cRNA probe, omission of cRNA probes and cross controls (mix of one antisense cRNA probe of interest with another sense cRNA probe and detection using the double or triple detection protocols) lacked unspecific staining. These results indicate that the antisense cRNA probes were specific and the detection systems did not create labeling artifacts.

For quantitative analyses of co-expression of NPY and 5-HTR mRNA in double ISH labelings, tissue sections spaced at least 30 μm apart were analyzed to rule out repeated evaluation of the same neuron. Per animal, at least nine cryosections from anterior, medial and posterior coronal section level of left and right La and BL and at least 35 neurons per nucleus were analyzed. Then, the percentage values per animal were subjected to statistical tests. To test the normal distribution of double ISH data we used the Shapiro–Wilk test and to analyze statistical differences in sex and nuclei, we applied a two-way ANOVA. Statistical analyses were carried out using the free software environment for statistical computing R, version 2.10.0 (http://cran.r-project.org/). For triple labelings, only qualitative evaluation was done.

## Results

### NPY, 5-HTT and 5-HT immunoreactivity: LM observations

**Fig. 1:**
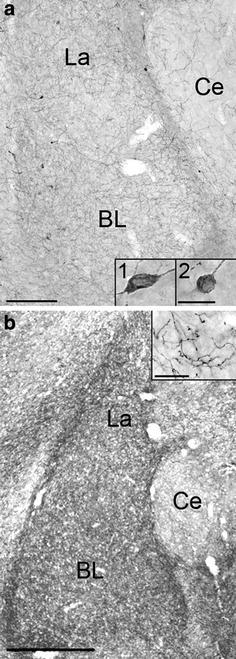
Light micrographs of NPY (**a**) and 5-HTT-immunoreactivities (**b**) in frontal sections through the rat amygdala at a mid-rostrocaudal level. *BL* basolateral nucleus, *Ce* central nucleus, *La* lateral nucleus. *Insets* in **a** show a fusiform (1) and a round (2) NPY-ir soma, *inset* in **b** shows a high magnification of narrow 5-HTT-ir afferents with irregularly spaced oblong varicosities in La. *Scale bars* 460 μm in **a** and **b**, 20 μm in all *insets*

**Fig. 2:**
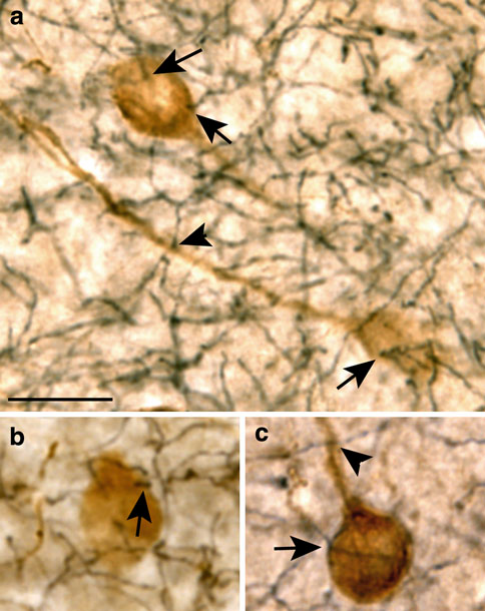
NPY-ir neurons (*brown*) displaying close appositions of 5-HTT-ir fibers (*black*) in BL. *Arrows and arrowheads* (**a–c**) point to 5-HTT-ir apparent contacts on NPY-ir somata and proximal processes, respectively. *Scale bar* in **a** is 20 μm and is also valid for **b** and **c**

Using single labeling, NPY-immunoreactivity in the Ce was scarce, but numerous fusiform or round NPY-ir somata (Fig. 1a, insets) and dense plexus of NPY-ir fibers were observed in the La and BL (Fig. 1a). Similarly, in 5-HTT single labeling studies, 5-HTT-ir fiber density in Ce was low (Fig. 1b) but the La and BL subregions exhibited a very dense plexus of narrow, smooth 5-HTT-ir axons with irregularly spaced oblong varicosities (Fig. 1b, inset). 5-HT labeling yielded comparable innervation patterns and fiber morphology, indicating that immunoreactivity for 5-HTT reliably labeled serotonergic fibers of this type (data not shown). Immunolabeling for 5-HT was generally less intense and somewhat more variable as compared to immunodetection of 5-HTT. In addition, a few axons characterized by a larger diameter and greater varicosity size were detected using immunolabeling for 5-HT (not shown). Fibers of this type were generally rare in the amygdala, were absent in La and very scarce in BL, and were never found in close vicinity to NPY-ir somata in BL. Based on the LM analysis of morphological interrelations of serotonergic afferents and NPY-ir somata, quantitative analyses were carried out using dual labeling of NPY and 5-HTT, which yielded specific and consistent results of numerous contacts between serotonergic fibers and NPY-ir neurons (Fig. 2). In La, 98.4 % (SD: ±2.6; *N*_animals_ = 6; *n*_neurons_ = 177) of all NPY-ir neurons displayed peri-somatic appositions of 5-HTT-ir fibers, and 2.1 (SD: ±0.3) appositions per soma were counted, on average. In BL, 95.3 % (SD: ±6.5; *N*_animals_ = 6; *n*_neurons_ = 102) of all NPY-ir neurons displayed peri-somatic appositions of 5-HTT-ir fibers, and 2.4 (SD: ±0.5) appositions per soma were counted, on average (Fig. 2a, b). 5-HTT-ir fibers running close to the NPY-ir somata for some distance were frequently observed in both nuclei (Fig. 2c). Moreover, appositions of 5-HTT-ir fibers on proximal NPY-ir processes were often found (Fig. 2a, c). Due to the difficulty in consistently identifying NPY-labeling in proximal processes caused by the partially weak reaction product in dual-labeled tissue, quantitative analysis of contacts onto dendrites was not carried out. Separate evaluation of appositions on fusiform and round NPY-ir somata yielded no differences.

### NPY and 5-HT immunoreactivity: EM observations

**Fig. 3:**
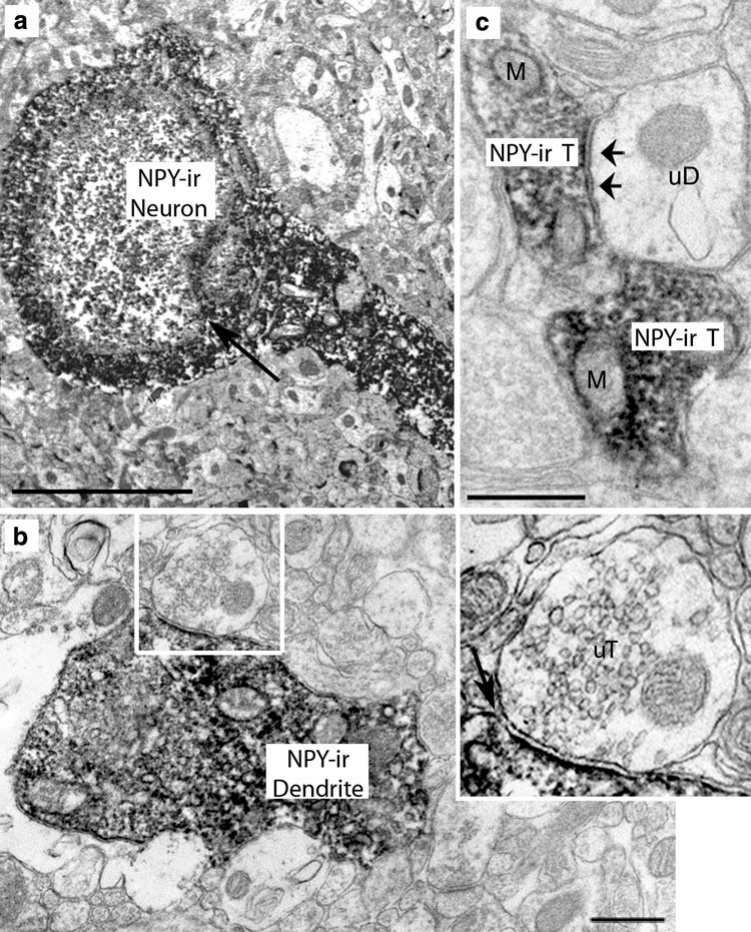
NPY-immunoreactive profiles in the La at the ultrastructural level. NPY-immunoreactivity is localized in cell bodies frequently displaying indented nuclei (*arrow* in **a**), in dendrites (**b**) synaptically contacted by unlabeled terminals (*inset* represents magnification of *boxed area* in **b**; *arrow points* at synaptic cleft) and in axon terminals forming symmetric synapses (**c** *arrows point* at postsynaptic area in a small unlabeled dendrite). *M* mitochondrion, *uD* unlabeled dendrite, *uT* unlabeled terminal. *Scale bar* in **a** is 5,000 nm, in **b** and **c** 500 nm

**Fig. 4:**
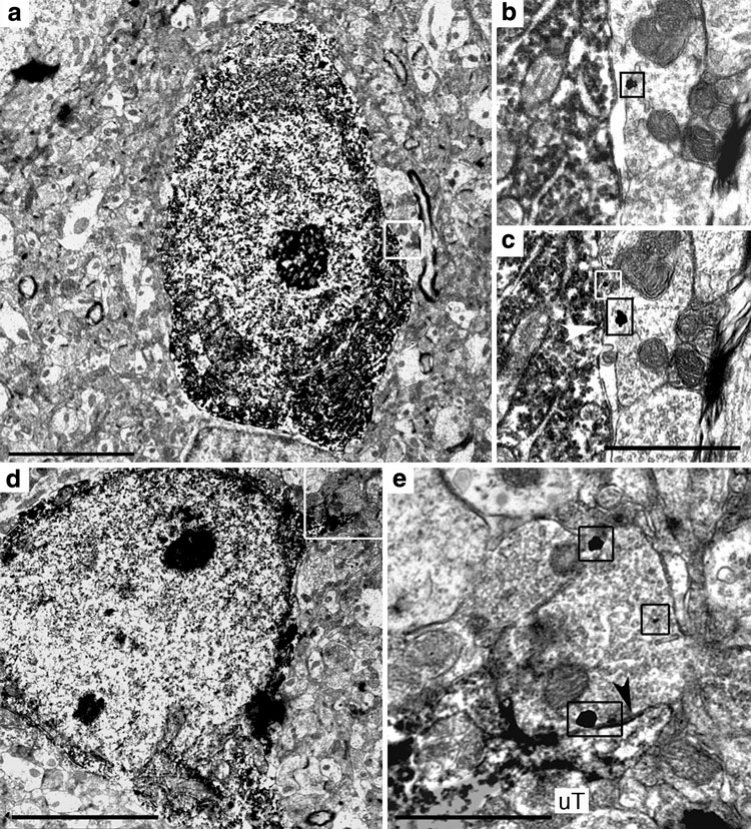
Interrelations between 5-HT-ir axon terminals and NPY-ir somata in La and BL. **a** A fusiform NPY-ir soma displaying a direct membrane apposition with a 5-HT-ir axon terminal. **b**, **c** Serial sections of *boxed area* in **a** at higher magnification. *Small boxes* frame immunogold-silver particles located within the 5-HT-ir axon terminal; *arrowhead* indicates the membrane contact. **d** A round NPY-ir soma whose somatic spine receives a symmetric-type synaptic contact from a 5-HT-ir axon terminal (*boxed area*) shown at higher magnification in **e**. *Small boxes* in **e** frame immunogold-silver particles located within the 5-HT-ir axon terminal and *arrowhead* indicates the synaptic contact. *Scale bars* in **a** and **d** are 5,000 nm, in **c** 1,000 nm (also valid for **b**) and in **e** 1,000 nm

Since use of two primary antibodies raised in the same species (rb) was not suitable for the EM dual labeling procedure, a rat anti-5-HT antibody was utilized instead of rb anti-5-HTT for this portion of the study. At the ultrastructural level, NPY-immunoreactivity was specifically detected by chromogenic visualization of DAB within the cytoplasm of fusiform and round cell bodies. These possessed irregularly formed and frequently indented nuclei (Figs. 3, 4). The immunoperoxidase reaction product was also found within proximal dendrites (Fig. 3a, b) that received synaptic contacts from unlabeled axon terminals (uT; Fig. 3b). Moreover, axon terminals containing NPY frequently formed symmetric synapses with unlabeled dendrites (uD; Fig. 3c) and unlabeled somata (data not shown). Asymmetric contacts were not observed between NPY-ir axon terminals and postsynaptic structures. In general, NPY-ir axon terminals were densely packed with synaptic vesicles and exhibited diffusely distributed DAB reaction product. In mitochondria, no DAB reaction product was observed (Fig. 3c). 5-HT-ir terminals exhibited densely packed, small round vesicles and formed mostly symmetric-type synaptic contacts with target structures (Fig. 4b, c, e). The EM approach confirmed the LM analysis of dual immunoreactions. Using serial section analysis, direct membrane appositions of 5-HT-ir axons on round and fusiform NPY-ir cell bodies and small symmetric-type synaptic contacts were observed, in one case on a small somatic spine (Fig. 4a–e).

### 5-HTR mRNA expression of NPY mRNA-reactive neurons

**Fig. 5:**
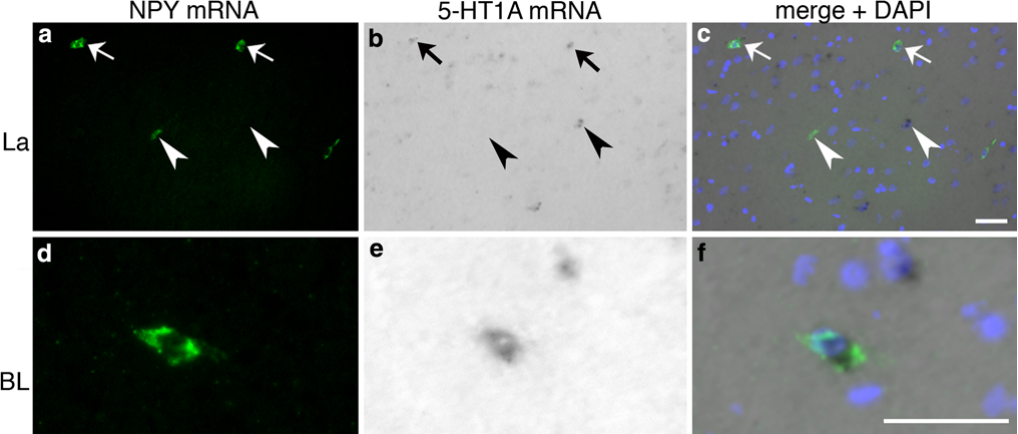
Double ISH (FISH/CISH) for NPY and 5-HT1A mRNA in La (**a–c**) and BL (**d–f**). *Arrows* indicate co-expression of both mRNAs in (**a–c**). *Arrowheads* in (**a**–**c**) point to cells single labeled for NPY and 5-HT1A mRNA, respectively. *Scale bar* in **c** (50 µm) is also valid for **a** and **b**, *scale bar* in **f** (50 µm) is also valid for **d** and **e**

**Fig. 6:**
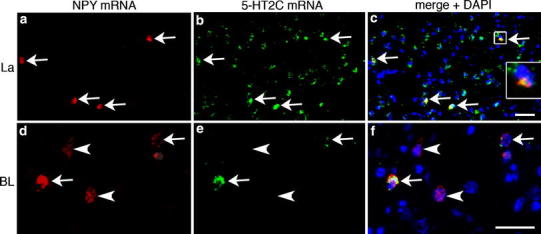
Double ISH (double FISH) for NPY and 5-HT2C mRNA in La (**a–c**) and BL (**d–f**). *Arrows* and *inset* in **c** and **f** indicate co-expression of both mRNAs. *Arrowheads* in **d**–**f** point to single-labeled NPY mRNA-reactive cells. *Scale bar* in **c** (50 µm) is also valid for **a** and **b**, *scale bar* in **f** (50 µm) is also valid for **d** and **e**

**Fig. 7:**
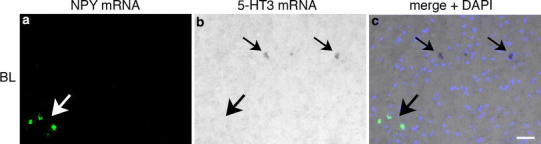
Double ISH (FISH/CISH) for NPY and 5-HT3 mRNA in BL. No co-expression of NPY and 5-HT3 mRNA was observed. *Large arrows point* to single-labeled NPY mRNA-reactive neurons, *small arrows* to single-labeled 5-HT3 mRNA-reactive neurons. *Scale bar* in **c** (50 µm) is also valid for **a** and **b**

**Fig. 8:**
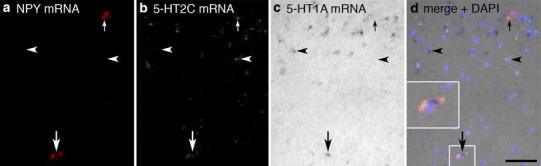
Triple ISH for NPY, 5-HT1A and 5-HT2C mRNA in BL. Co-expression of NPY with 5-HT1A and 5-HT2C was found in individual neurons (*large arrows*, *inset* in **d**). *Small arrows point* to a neuron double labeled for NPY and 5-HT2C mRNA, *arrowheads* indicate single-labeled cells for 5-HT1A and 5-HT2C mRNA. *Scale bar* in **d** (50 µm) is also valid for **a**–**c**

**Fig. 9:**
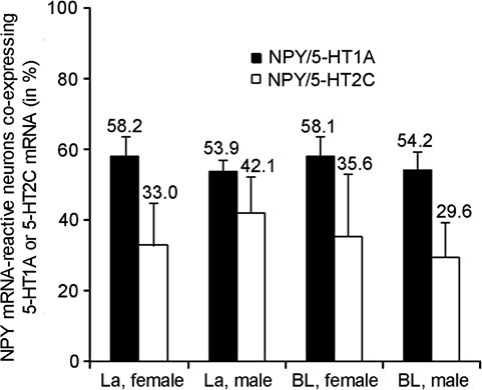
Co-expression of NPY mRNA with 5-HT1A and 5-HT2C mRNA. Data are presented as arithmetic mean + standard deviation. The *numbers above the bars* indicate percentage values of co-expression. *BL* basolateral nucleus, *La* lateral nucleus

For all mRNAs, single CISH showed a labeling pattern similar to that seen in previous ISH studies (Bonn et al. 2012; Mengod et al. 1990; Miquel et al. 1991; Tecott et al. 1993). Cells displaying varying levels of reactivity for NPY mRNA were scattered throughout La and BL (Figs. 5, 6, 7, 8). Many lightly and few moderately 5-HT1A mRNA-reactive cells were found in both nuclei (Figs. 5, 8). 5-HT2C mRNA-reactive cells were numerous with comparatively intense reactivity in La (Fig. 6b) and were also frequently found but with lower reaction intensity in BL (Fig. 6e). Moderate 5-HT3 mRNA-reactivity was observed in scattered cells in both nuclei (Fig. 7). Quantitative analyses of double ISH for NPY and 5-HTR mRNAs showed that in La and BL of female rats, 58.2 and 58.1 %, respectively, of NPY mRNA-reactive neurons co-expressed 5-HT1A mRNA, in male rats the percentage was 53.9 and 54.2 %, respectively (Figs. 5, 9). In La and BL of females, 33.0 and 35.6 %, respectively, of NPY mRNA-reactive neurons co-expressed 5-HT2C mRNA; in males it was 42.1 and 29.6 %, respectively (Figs. 6, 9). Inter-individual differences in percentages as represented by the standard deviations of arithmetic means were much higher for NPY/5-HT2C than for NPY/5-HT1A co-expression for both sexes in both nuclei, particularly in the BL of females (Fig. 9). The results of corresponding two-way ANOVA showed no significant differences for co-expression of the respective 5-HTR in NPY-producing neurons between nuclei or gender. No co-expression of NPY and 5-HT3 mRNA was observed in La and BL of rats of both sexes (Fig. 7). In FISH/CISH triple labelings, co-expression of NPY with 5-HT1A and 5-HT2C mRNA was detected in individual cells in La and BL (Fig. 8). In addition, double-labeled neurons co-expressing NPY and 5-HT2C mRNA (Fig. 8) or NPY and 5-HT1A (not shown), and neurons single labeled for 5-HT1A or 5-HT2C mRNA (Fig. 8) and for NPY mRNA (not shown) were observed in the triple labeled preparations.

## Discussion

The LM analyses of the present study suggest that virtually all NPY-ir neurons in La and BL receive peri-somatic serotonergic innervation. Direct membrane appositions and small symmetric-type synaptic contacts between serotonergic afferents and NPY-ir neurons were verified by ultrastructural analysis. The intense peri-somatic serotonergic innervation indicates a substantial direct impact of serotonergic innervation on NPY neurons, since neurotransmission at the soma is in a position to prominently influence neuronal function. Our multiple ISH analyses show that NPY mRNA-reactive neurons in the La and BL differentially express 5-HT1A and/or 5-HT2C mRNA indicating diverse effects of 5-HT on this interneuronal subpopulation. 5-HT3 mRNA expression was not observed in NPY-producing neurons.

### Methodological considerations

5-HTT has frequently been used as a reliable and specific marker for serotonergic afferents in different brain regions (Brown and Molliver 2000; Eliava et al. 2003; Parent et al. 2010). Accordingly, our immunolabeling of serial sections using 5-HT and 5-HTT antibodies showed similar patterns of innervation in La and BL, with virtually identical characteristics of innervation density and fiber morphology corresponding to earlier findings (e.g. Fallon and Ciofi 1992; Muller et al. 2007b; Vertes et al. 1999). Both reactions labeled dense plexus of narrow serotonergic fibers in La and BL presumably originating in the DR (Brown and Molliver 2000; Kosofsky and Molliver 1987; Mamounas et al. 1991). Thicker beaded fibers representing serotonergic afferents from the median raphe (Kosofsky and Molliver 1987), which supposedly lack 5-HTT (Brown and Molliver 2000), were detected in 5-HT-immunolabeling only, were not observed in the La and only very rarely found in the BL, in accordance with findings for median raphe afferents to the amygdala (Vertes et al. 1999).

Patterns of NPY-immunoreactivity and morphology of NPY-ir neurons in La and BL were consistent with the existing literature (Gustafson et al. 1986; Quidt de and Emson 1986). In dual labeling studies, NPY-immunolabeling provided detection of neuronal somata but did not always reliably label dendrites. Therefore, quantitative analyses were restricted to peri-somatic contacts between serotonergic afferents and NPY-ir neurons. 5-HT/NPY double immunoelectron microscopic preparations served to prove direct membrane contacts and synapses between serotonergic afferents and NPY-ir somata. 5-HT- and NPY-immunoreactivity was localized using a combined pre-embedding immunoperoxidase and immunogold detection method. This technique provides high resolution subcellular localization of the antigens while preserving ultrastructural morphology. However, pre-embedding immunolabeling in vibratome sections can produce limited reagent penetration, a caveat with this approach. In order to minimize the problems associated with poor penetration, we collected ultrathin sections near the tissue-Epon interface where penetration of the antibody is optimal to ensure that both immunolabels were clearly detectable in sections included in the analysis.

### Serotonergic innervation of NPY-ir interneurons: morphological characteristics and functional implications

Our analyses indicated that NPY neurons in La and BL possessed either round or fusiform cell bodies. NPY-ir La and BL neurons represent a subpopulation of somatostatinergic neurons, for which an ovoid to fusiform perikaryal morphology was described (McDonald 1989). Recent cell classification according to multifunctional parameters using cluster analyses additionally documented that La NPY-ir neurons belong to electrophysiologically and morphologically distinct subclasses of interneurons (Sosulina et al. 2006). Our LM data show that virtually all fusiform and round NPY-ir somata in the La and BL are contacted by serotonergic fibers with the morphology proposed for DR afferents, and most somata were targeted by more than one serotonergic fiber. The ultrastructural data provide compelling evidence that this important interneuron subgroup is a direct target of serotonergic innervation.

Previous quantitative ultrastructural studies have documented that 89 % of SOM-ir terminals innervating pyramidal cells contact distal dendrites and spines, and that about 15 % of all terminals synapsed with other interneurons (Muller et al. 2007a). Since virtually all NPY-ir interneurons are also somatostatinergic with about 30 % (BL) and 80 % (La) of SOM-ir neurons co-expressing NPY (McDonald 1989), it is likely that distal pyramidal cell dendrites are preferential targets also of NPY-ir terminals. Previous documentation of presumably symmetric synaptic contacts of NPY-ir terminals on BL pyramidal cell dendritic structures (Cui et al. 2008), and our findings of exclusively symmetric synaptic contacts of NPY-ir terminals and of small dendrites as postsynaptic targets support this suggestion. Recent electrophysiological and behavioral data provide compelling support for an inhibitory effect of NPY on pyramidal cells in the basolateral amygdala, mediated via Y1 NPY receptors (Giesbrecht et al. 2010; Sosulina et al. 2008; Thorsell 2010). Thus, NPY-producing interneurons may be in a position to inhibit the propagation of excitatory inputs onto spines and distal dendrites to the pyramidal cell body. On the other hand, NPY-ir neurons might also target inhibitory interneurons, as has been documented for other subgroups of interneurons (Muller et al. 2005, 2007a). Quantitative ultrastructural analyses, which will be required to conclusively document the exact synaptology of NPY-ir La and BL interneurons, are complicated by the fact that NPY-immunoreactivity may be present in extrinsic afferents (e.g. Cui et al. 2008; Truitt et al. 2009), albeit in some of these at a very low level (Asan 1998; Gustafson et al. 1986). In any case, the anxiolytic effect of NPY in the amygdala is linked to the function of NPY-ir interneurons, since the number of these neurons is correlated closely with anxiety-like behavior (Truitt et al. 2009; Yilmazer-Hanke et al. 2002, 2004).

Serotonergic afferents to the basolateral amygdaloid complex originate mostly in the dorsal part of the mid-rostrocaudal region of the DR (Lowry et al. 2005). This DR region is activated by different stress paradigms and anxiety-related stimuli, including anxiogenic drugs (Abrams et al. 2005; Amat et al. 1998; Gardner et al. 2005; Grahn et al. 1999). Accordingly, behavioral studies using microdialysis to assess 5-HT concentrations in the BL showed an increase in 5-HT release during stress and anxiety states (Kawahara et al. 1993; Rueter and Jacobs 1996; Zanoveli et al. 2009). Our morphological findings indicate that these stress- and anxiety-responsive serotonergic afferents may be in a position to influence the activity of NPY-ir interneurons and thus may modulate an important intrinsic anxiolytic inhibitory network of the La and BL.

### Serotonin receptor expression of NPY-producing neurons

To date, 5-HTR expression of NPY-producing amygdaloid interneurons has not been studied. Our findings document that more than half of NPY mRNA-reactive interneurons in the La and BL co-express 5-HT1A and between 30 and 40 % co-express 5-HT2C mRNA. 5-HT1A- and 5-HT2C-expressing NPY-producing neurons apparently represent largely separate populations with individual neurons co-expressing both receptors and a few cells not expressing either. Whether the 5-HT1A- and 5-HT2C-expressing neurons represent the two morphological subclasses of NPY neurons with round or fusiform cell bodies, or perhaps differ with respect to their postsynaptic targets, remains to be determined. Furthermore, while the present results document that NPY mRNA-reactive neurons are capable of producing the respective receptors, the responsivity of the neurons to serotonergic input depends on further parameters, such as mRNA editing of 5-HT2C (Werry et al. 2008), the actual level of receptor proteins, the subcellular localization, membrane insertion and desensitization processes, and expression of possible additional 5-HTR (see also below). For 5-HT3, we documented that this receptor is not expressed in NPY-producing neurons, a finding consistent with earlier observations that 5-HT3-ir neurons do not overlap with somatostatinergic neurons in the La and BL (Mascagni and McDonald 2007).

The proportions of NPY mRNA-reactive neurons expressing 5-HT1A and 5-HT2C did not differ between La and BL, indicating that serotonergic influence is similar in both nuclei. Moreover, sex differences were lacking in both nuclei. However, the inter-individual variations were large between animals for NPY/5-HT2C mRNA co-expression, particularly in the BL of female animals. This might indicate that 5-HT2C expression is subject to extrinsic influence—a possible mechanism for modulation of the 5-HT responsiveness of NPY-producing neurons, for instance by gonadal or adrenocortical stress hormones. In female rats, 5-HTR levels in the amygdala and other brain regions are regulated by estradiol (Biegon and McEwen 1981). Sex differences in 5-HT transmission parameters and anxiety-like behavior are frequent (e.g. Duchesne et al. 2009; Mitsushima et al. 2006), and can easily be addressed in future experimental studies.

### Serotonin innervation and receptor expression of identified La and BL neurons: implications for anxiety and stress

The excitability of pyramidal cells in the La and BL, which presumably represents a functional basis of anxiety states, is potently controlled by inhibitory GABAergic interneurons (Lang and Pare 1997, 1998; Rainnie et al. 1991; Washburn and Moises 1992). Serotonergic modulation of intrinsic inhibitory circuits in these nuclei, therefore, appears of particular importance for adequate emotionality, and dysfunction of inhibitory control might underlie clinically relevant behavioral alterations associated with stress and anxiety disorders. The dense serotonergic fiber plexus of the La and BL have been shown to form numerous contacts with pyramidal cells and different types of interneurons (Muller et al. 2007b). Morphological characteristics of contacts on other interneurons were similar to those documented for serotonergic innervation of NPY-ir neurons in the present study. Thus, our findings complement the previous findings, indicating serotonergic input to diverse interneuronal subpopulations in these nuclei, including the small but functionally important population of NPY-ir interneurons.

Numerous pharmacobehavioral studies in rodents have indicated that serotonergic transmission via the 5-HT1A, 2 and 3 receptor subtypes in the basolateral amygdala is of crucial importance for serotonergic impact on anxiety-like behavior, particularly following exposure to different stress or fear conditioning paradigms (Campbell and Merchant 2003; Christianson et al. 2010; Gonzalez et al. 1999; Li et al. 2012; Menard and Treit 1999; Morrison and Cooper 2012; Nevins and Anthony 1994). 5-HT1A and 5-HT2 are G protein-coupled receptors, but while 5-HT1A activation mediates inhibitory membrane hyperpolarization by opening G protein-coupled inwardly rectifying potassium channels (Reuveny et al. 1994), 5-HT2 activation increases the firing rate of neurons (Barnes and Sharp 1999; Rainnie 1999), with 5-HT2C presumably facilitating excitatory membrane depolarization by closing two-pore domain potassium channels, especially TASK-3 (Weber et al. 2008). 5-HT3 is the only ionotropic 5-HTR, a cation-selective ion channel (Barnes and Sharp 1999). While 5-HT3 activation in the amygdala appears to exert mostly anxiogenic effects (Mascagni and McDonald 2007), evidence for both anxiogenic and anxiolytic effects of modulating 5-HT1A and 5-HT2 receptor activation has been given (Menard and Treit 1999). The majority of recent findings points to primarily anxiolytic actions of 5-HT1A and anxiogenic actions of 5-HT2C (Christianson et al. 2010; Leite-Panissi et al. 2006; Li et al. 2012; Morrison and Cooper 2012; Vicente and Zangrossi 2011).

In our ISH studies, we found 5-HT1A and 5-HT2C mRNA in numerous cells of the La and BL in addition to NPY-producing cells. 5-HT1A and 5-HT2A have been localized to both pyramidal cells and PV-producing inhibitory interneurons in the BL (Aznar et al. 2003; McDonald and Mascagni 2007). It has not been studied yet whether the different receptors are localized in the same cells or in separate subgroups. 5-HT2A was detected additionally in a subpopulation of SOM-ir interneurons (McDonald and Mascagni 2007), but whether this subpopulation overlaps with that of NPY-producing somatostatinergic interneurons has not been determined. The identity of the strongly and moderately 5-HT2C mRNA-reactive NPY mRNA-negative cells observed in our ISH investigation in the La and BL, respectively, is unclear, since the cellular expression of 5-HT2C in the amygdala has not been studied yet. However, the high frequency of these cells in the La makes a production of this receptor in pyramidal cells likely.

In view of the expression of inhibitory and excitatory 5-HTR in both pyramidal cells and inhibitory interneurons, interpretation of the pharmacobehavioral findings is not trivial. Rodriguez-Manzanares and co-workers (2005) showed that stress, which is accompanied by elevated 5-HT concentration in the basolateral amygdala (Amat et al. 1998; Rueter and Jacobs 1996), dampens the inhibitory control of GABAergic interneurons facilitating pyramidal cell activation and the formation of fear memory. 5-HT1A-mediated serotonergic inhibition of strongly inhibitory NPY neurons which target pyramidal cells might well be an element of this process. On the other hand, continuous suppression of GABAergic inhibitory control would lead to hyperexcitation of the basolateral amygdala, a state often considered as a correlate of increased, mostly negative, emotional state as indicated by increased anxiety or anxiety-like behavior in animals (Isoardi et al. 2007; Rosen and Schulkin 1998; Rosenkranz et al. 2010; Zhou et al. 2010). Based on various experimental evidence, it has been suggested that 5-HT2A receptor activation in PV-producing interneurons enhances inhibition of pyramidal cells, resulting in a reduction of output to circuits mediating anxiety-related behavior (Hale et al. 2010; Rainnie 1999). Serotonergic excitation of the subgroup of 5-HT2C-expressing NPY neurons might contribute to enhanced pyramidal cell inhibition. The proposed functions of 5-HT/NPY interaction via the different receptors require a temporal sequence of 5-HT-mediated effects, perhaps caused by desensitization mechanisms or other differential modulation of receptor functions (Snoeren et al. 2011), which might also provide the basis for functions of NPY-producing neurons expressing both 5-HTR subtypes. However, the suggestion of a net inhibitory effect of 5-HT2C-mediated excitation of NPY-producing interneurons is difficult to reconcile with the predominantly anxiogenic properties of 5-HT2C agonists in the BL (Christianson et al. 2010; Li et al. 2012). Further morphological and electrophysiological experiments are required to test the different hypotheses.

In conclusion, our findings suggest that 5-HT/NPY interactions contribute to the complicated network of intrinsic inhibitory circuits in the basolateral amygdala, a nodal structure for modulating anxiety-related behavior, emotional learning and memory formation (Lowry et al. 2005; Roozendaal et al. 2009). Evidence in support of differential receptor expression in the small but functionally important inhibitory NPY-producing interneuron population suggests diverse actions of 5-HT, supporting the relevance of serotonergic transmission in the La and BL for adjusting behavior and cognitive functions to changing environmental demands.
